# Effect of benzoic acid surface modified alumina nanoparticles on the mechanical properties and crystallization behavior of isotactic polypropylene nanocomposites†

**DOI:** 10.1039/c8ra01069b

**Published:** 2018-06-06

**Authors:** Xiaofeng Jiang, Wenxue Zhang, Shicheng Zhao, Shuai Zhou, YaoQi Shi, Zhong Xin

**Affiliations:** Shanghai Key Laboratory of Multiphase Materials Chemical Engineering, State-Key Laboratory of Chemical Engineering, East China University of Science and Technology Shanghai 200237 China xzh@ecust.edu.cn; Lanzhou Petrochemical Research Center PetroChina 730060 China

## Abstract

The effect of benzoic acid (BA) surface modified alumina (Al_2_O_3_) nanoparticles (NPs) on the mechanical properties and crystallization behavior of isotactic polypropylene (iPP) nanocomposites was studied. Characterization of the modified Al_2_O_3_ NPs (BA-Al_2_O_3_) by FTIR and XRD analyses confirmed that benzoic acid molecules chemisorb on the surface of the NPs, forming benzene groups-rich microstructures. A considerable increase in the tensile strength, flexural modulus, and toughness was observed for the nanocomposites with only 0.2 wt% BA-Al_2_O_3_. Enhanced interfacial adhesion with the matrix was achieved, which enabled effective reinforcement of the nanocomposites. The higher crystallization temperature along with shorter crystallization halftime indicated the higher nucleation activity of BA-Al_2_O_3_. Furthermore, the interchain conformational ordering of iPP was significantly accelerated in the presence of the BA-Al_2_O_3_ NPs. The CH–π interaction between the polymer and BA-Al_2_O_3_ NPs was considered to facilitate the attachment of the iPP chains and stimulate conformational ordering, crystallization, as well as mechanical properties of nanocomposites.

## Abstract 2

The CH–π interactions between polypropylene and functionalized alumina (BA-Al_2_O_3_) nanoparticles improve the mechanical performance and conformational ordering of nanocomposites.

## Graphical abstract

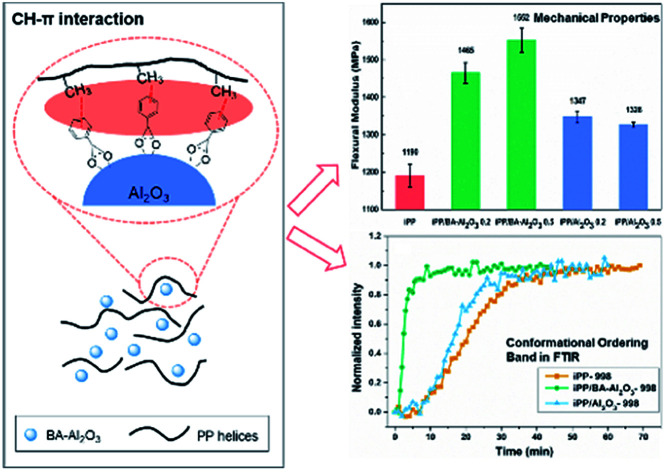

## 1. Introduction

Isostatic polypropylene (iPP) is one of the most widely used polymers for nanocomposite preparation due to its availability, ease of processing, and relatively low cost.^1–3^ A range of nanoparticles (NPs), such as those of montmorillonite (MMT),^4^ carbon nanotubes (CNTs),^5,6^ graphene,^7,8^ alumina,^9^ and layered double hydroxides (LDH),^10,11^ has been broadly studied as fillers for nanocomposites. Among the commonly used fillers, alumina (Al_2_O_3_) NPs have attracted particular attention due to their good thermal conductivity, high mechanical strength, low cost, and non-toxicity.^12,13^ However, the tendency to form aggregates and the poor dispersion in polymers have detrimental effects on the performance of the resulting nanocomposites. To inhibit unfavorable aggregation and enhance interfacial interactions, surface modification of the NPs has been employed as a useful and flexible strategy in nanocomposite technology.^14–16^

Extensive research has focused on the surface functionalization of Al_2_O_3_ NPs, such as coating with silane coupling agents^9,17,18^ and grafting with polymers.^19–21^ However, the modified Al_2_O_3_ NPs generally have no significant effect on the mechanical performances and crystallization behavior of non-polar polymer nanocomposites, particularly in low contents.^9,20,22–25^ Zhao and Li studied the crystallization behaviors of nanocomposites containing 1.5–5.0 wt% of Al_2_O_3_ (pretreated with a silane coupling agent), and the crystallization temperature of the nanocomposites was only enhanced by 4 °C.^9^ The Al_2_O_3_ NPs are generally surface-modified with hydrocarbon chain structures to improve the compatibility in PP and PE.^15,22,26^ Nonetheless, there is no special interfacial interaction between the PP backbone and the hydrocarbon chain structure of the modified Al_2_O_3_ NPs surface. Theoretical studies have demonstrated that the interfacial interaction between the adsorbing surface and the polymer is important to lower the thermodynamic potential for crystallization.^27,28^ Therefore, the study of functionalization of Al_2_O_3_ NPs with organic groups to generate a special interfacial interaction and subsequently improve the crystallization behavior at a low content is essential.

Recently, some studies have demonstrated that the CH–π interaction has a critical effect on the nucleation and crystallization of polymers.^29,30^ It is believed that this interaction lowers the free energy barrier for nucleation by modulating the segmental motion of the polymer and its subsequent crystallization and growth.^29,30^ However, few studies have focused on the functionalization of inorganic NPs with aromatic groups to generate CH–π interactions. Thus, we functionalized Al_2_O_3_ NPs with aromatic groups to form CH–π interactions with the alkyl groups of the iPP chain. Furthermore, the surface properties of the Al_2_O_3_ NPs could be tuned by different carboxylic acids.^31,32^ A few groups have reported the use of these acids as surface modifiers for advanced nanocomposites.^33,34^ Therefore, we investigated the effect of benzoic acid-modified Al_2_O_3_ NPs on the mechanical properties and crystallization behavior of iPP nanocomposites. Through an effective and simple method, aromatic groups-functionalized Al_2_O_3_ NPs were obtained. The specific interactions between iPP and the functionalized NPs improved the properties of the polymer at a low NP content.

Herein, we report the study of the mechanical properties and crystallization behavior of iPP/modified-Al_2_O_3_ nanocomposites. The Al_2_O_3_ NPs were modified with commercially available benzoic acid to tailor the surface chemistry of the NPs. Enhanced flexural, tensile, and impact properties implied the strong interfacial interaction between the iPP matrix and functionalized Al_2_O_3_ NPs (BA-Al_2_O_3_). A remarkable increase in the crystallization temperature was achieved at only 0.2 wt% NP content. Furthermore, time-resolved Fourier-transform infrared spectroscopy (FTIR) was employed to follow the intrachain conformation ordering and crystallization evolution of the nanocomposites. The CH–π interaction was considered to facilitate the crystallization behavior and mechanical properties of the iPP/BA-Al_2_O_3_ nanocomposites.

## 2. Experimental

### 2.1 Materials

The iPP sample (trade name T30S) was provided by Jiujiang Petroleum Chemical Co., China, with a melt flow rate of 2.9 g/10 min (230 °C/2.16 kg), *M*_w_ of 24.4 × 10^4^ g mol^−1^, and *M*_w_/*M*_n_ of 4.05. The γ-alumina (Al_2_O_3_) NPs with an average diameter of 10 nm, used in this study, were provided by Adamas Reagent Co., Ltd. Benzoic acid (BA), toluene, and 2-propanol were purchased from Shanghai Lingfeng Chemical Corp. Ethanol was obtained from Shanghai Titan Science Corp. All chemicals were of analytical grade and used without further purification.

### 2.2 Synthesis of carboxylate-functionalized nanoparticles

**Fig. 1:**
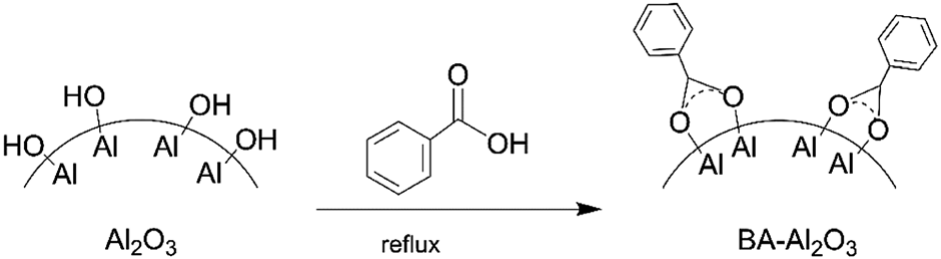
Schematic diagram of the preparation of functionalized Al_2_O_3_ NPs.

The functionalization of Al_2_O_3_ NPs is shown in Fig. 1. According to reported procedures,^32^ the alumina nanoparticles (6 g) were refluxed overnight in toluene (100 mL) and benzoic acid (5.27 g). The functionalized particles were then centrifuged for 30 min and re-dispersed in 2-propanol (2 × 30 mL) and ethanol (1 × 30 mL), and then centrifuged again to remove unreacted carboxylic acid. Finally, the functionalized NPs (BA-Al_2_O_3_) were oven-dried at 80 °C overnight.

### 2.3 Characterization of nanoparticles

Fourier-transform infrared (FT-IR) spectroscopy was performed to study the interaction between BA and Al_2_O_3_ NPs. The FT-IR spectra with a resolution of 4 cm^−1^ were collected using a Nicolet 5700 FTIR spectrometer with samples in KBr pellets.

Powder X-ray diffraction (XRD) patterns were recorded on a Bruker advanced D8 powder X-ray diffractometer (Cu Kα, *λ* = 1.5418 Å, 40 kV, 40 mA). The wide-angle scan ranged from 10–80° with steps of 0.02° and 0.1 s for each step.

Thermogravimetric analysis (TGA) experiments were conducted on a TA Instrument SDT Q600. The samples were run in an open alumina crucible under continuous air flow. The heating profile was equilibrated at 50 °C and then ramped at 10 °C min^−1^.

### 2.4 Nanocomposites preparation

The nanocomposites were prepared by mixing appropriate amounts of NPs and antioxidant (Irganox 1010 and 168, 0.1 wt% relative to the iPP powder, respectively), and subsequently melting the blend, followed by the injection-molding of tensile, flexural, and Izod impact bars. In our study, the nanocomposites with pristine Al_2_O_3_ NPs and BA-Al_2_O_3_ NPs were prepared for comparison. The compound was dry-blended by a high-speed mixer for 5 min. Then, the mixture was extruded by a twin-screw extruder (SJSH-30, Nanjing Rubber and Plastics Machinery Plant Co.) through a strand die and pelletized. The pellets were mold into standard test specimens by an injection-molding machine (CJ-80E, Guangdong Zhende Plastics Machinery Plant Co.). Standard dumbbell-shaped samples were produced for the tensile property test according to ASTM D-638 (type I). The samples for the flexural property test were rectangular with dimensions of 127 × 12.7 × 3.2 mm^3^ (ASTM D-790). Rectangular samples (63.5 × 12.7 × 6.4 mm^3^) with ‘V’ notches were produced according to ASTM D-256 for the impact strength test. The samples were denoted as iPP/BA-Al_2_O_3_*x*, where ‘*x*’ is the weight percentage of BA-Al_2_O_3_ (*x* wt%) relative to the weight of the iPP, *e.g.*, iPP/BA-Al_2_O_3_ 0.2.

### 2.5 Characterization of nanocomposites

The mechanical properties of the nanocomposites were studied according to the ASTM test methods, such as D-638 for tensile strength and D-790 for flexural modulus, using a universal testing machine (Shanghai D and G Measure Instrument Co.). The Izod impact strength was tested according to D-256 using an impact tester (Chengde Precision Tester Co., model I02.75). The reported values of the mechanical properties were averaged from seven independent measurements.

The DSC measurements of the nanocomposites were carried out on a TA Instruments Q2000 under nitrogen flow, which was calibrated with indium as the standard. For non-isothermal crystallization, the samples (3–5 mg) were first annealed at 200 °C for 5 min to erase any thermal history and subsequently cooled to 50 °C at a cooling rate of 20 °C min^−1^. Then, the samples were heated to 200 °C at a rate of 10 °C min^−1^. For isothermal crystallization, the samples were annealed at 200 °C for 5 min to eliminate the thermal history, cooled to the desired crystallization temperature (*T*_C_) at a cooling rate of 50 °C min^−1^, and maintained at *T*_C_ until crystallization was completed. The exothermal plots were recorded for subsequent data analysis.

The morphology studies of the pure iPP and nanocomposites were performed with an Olympus BX51 (Japan) polarized optical microscope (POM) attached with a DP70 digital camera and a THMS600 hot-stage. The extruded samples were placed between two microscopy slides, melted, pressed at 200 °C for 5 min to remove any trace of crystals, and then cooled to 139 °C at a cooling rate of 50 °C min^−1^ and maintained at 139 °C until crystallization was completed. Photographs were automatically taken at 1 min intervals.

The time-resolved FTIR measurements were performed on a Nicolet 5700 FTIR spectrometer (Thermal Scientific, USA) equipped with a heated transmission cell (Mettler FP82 hot stage). The films of pristine iPP and the nanocomposites were deposited on a KBr pellet to adopt the transmission mode over the wavenumber range of 400–4000 cm^−1^. The spectra were obtained by averaging 16 scans at resolution of 4 cm^−1^ with a 30 s interval and subtracting from the background spectra. Each sample was maintained at 200 °C for 5 min to erase any thermal history and cooled to 139 °C at a rate of 50 °C min^−1^. When the temperature reached 139 °C, the data collection started until the end of the crystallization. Herein, the intensity refers to the peak height of the characteristic bands, and the base lines are corrected for each spectrum to the same standard.^35^

## 3. Results

### 3.1 Characterization of the modified Al_2_O_3_

**Fig. 2:**
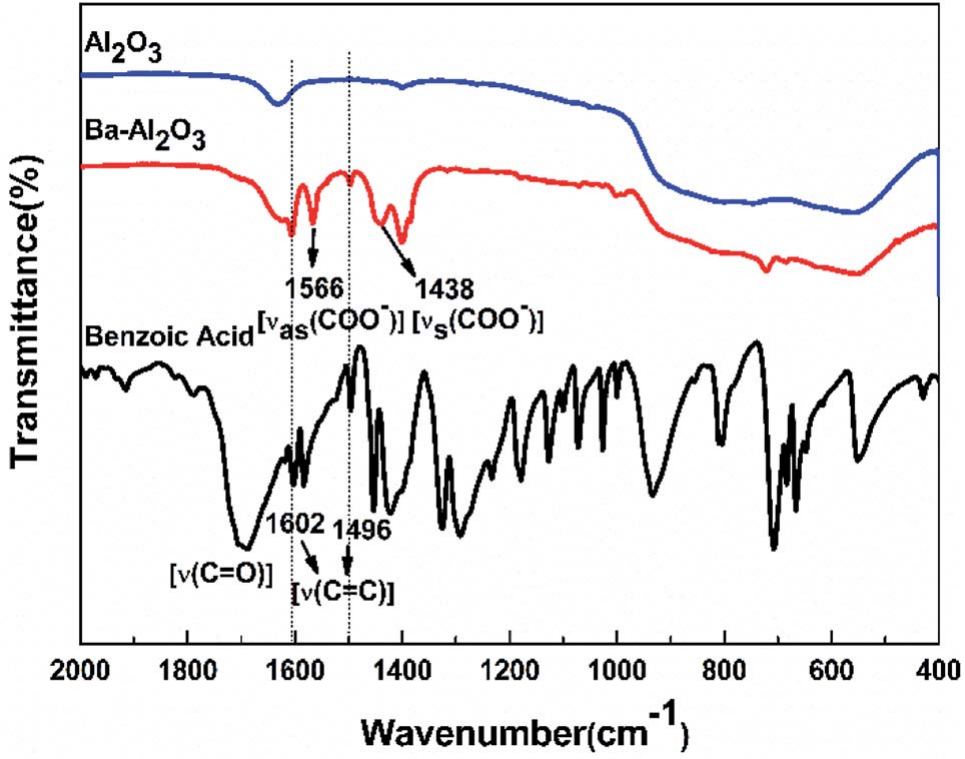
FTIR spectra of pristine alumina NPs, functionalized alumina NPs, and native carboxylic acids.

The FTIR spectra of the carboxylic acid-functionalized NPs BA-Al_2_O_3_ (Fig. 2) confirm the covalent attachment of the carboxylate moieties. The carbonyl band (C=O) of the free benzoic acid groups at 1690 cm^−1^ disappears, indicating the absence of unreacted acid in the products. After surface modification, the new bands at 1400 and 1600 cm^−1^ are assigned to the symmetric and asymmetric stretching of the carboxylate group (COO^−^).^33,36^ It is known from the previous investigations that interactions between carboxylate groups and metal atoms have been classified as monodentate, bridging bidentate, chelating bidentate, and ionic interactions.^33,37^ The relative wavenumber separation Δ between the asymmetric and symmetric carboxylate stretching bands can be used to distinguish these interactions.^33,37^ The largest Δ (200–320 cm^−1^) corresponds to monodentate interactions, and the smallest Δ (<110 cm^−1^) corresponds to chelating bidentate interactions. The medium range Δ (140–190 cm^−1^) corresponds to bridging bidentate interactions.^37^ In the present study, Δ has a value of ∼130 cm^−1^ (1566 − 1438 = 128 cm^−1^), suggesting that the interaction between the COO groups and Al atoms corresponds to a bridging bidentate bond. Concomitant with these changes is the appearance of the peaks at 1602 cm^−1^ and 1496 cm^−1^ ascribed to the stretching vibrations of the benzene ring. These data clearly reveal that benzene groups are covalently bonded to the Al_2_O_3_ NPs surface, as shown in Fig. 1.

The TGA plots of the pristine Al_2_O_3_, BA-Al_2_O_3_ NPs, and native benzoic acid are shown in ESI Fig. S1.† The pristine Al_2_O_3_ NPs show a weight loss of ∼3% due to degassing and volatilization of water or residual solvents.^38^ Alternatively, in the 200–500 °C temperature range, the significant weight loss of the BA-Al_2_O_3_ NPs is attributed to the thermal decomposition of the covalent modifications. It should be mentioned that the extruding and modeling temperature is 200 °C, which is below the decomposition temperature of functionalized alumina. Thus, the BA-Al_2_O_3_ NPs will be stable during the decomposition.

A comparison of the crystal structure of γ-Al_2_O_3_ before and after surface modification is presented in ESI Fig. S2.† In this profile, (311) at 2*θ* = 37.6°, (400) at 45.9°, and (440) at 67.0° are the principal reflections of γ-Al_2_O_3_. As shown in the figure, the peak position of the crystal plane does not shift after reaction with benzoic acid. This indicates that the typical pattern of γ-Al_2_O_3_ is not affected by the addition of carboxylic acid. We can suppose that the benzoic acid molecules are only chemisorbed on the surface of the NPs and are covalently attached on the surface, which does not change the crystal structure.

### 3.2 Mechanical properties of the nanocomposites

**Fig. 3:**
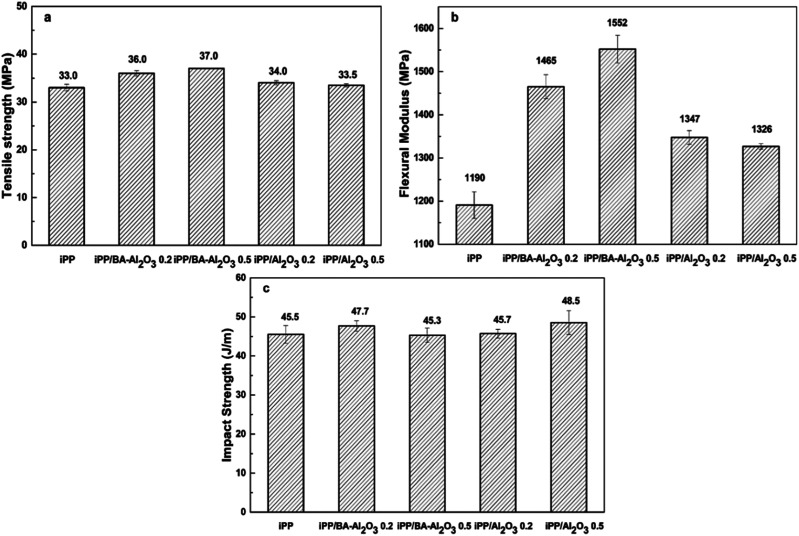
Influence of different nanoparticles on the (a) tensile strength, (b) flexural modulus, and (c) impact strength of iPP.

From the perspective of industrial application, it is important to investigate the effect of NPs on the mechanical properties of the nanocomposites. Fig. 3 shows the comparison of the influence of Al_2_O_3_ and BA-Al_2_O_3_ on the mechanical properties of the iPP nanocomposites. The addition of Al_2_O_3_ slightly improved the tensile strength and flexural modulus. However, the introduction of BA-Al_2_O_3_ causes a greater improvement in the tensile strength, flexural modulus, and Izod impact strength as compared to pristine Al_2_O_3_. The flexural modulus is increased by about 13.2% in the case of the as-received Al_2_O_3_ at content of 0.2 wt% as shown in Fig. 3b. However, in case of BA-Al_2_O_3_, at the same content of 0.2 wt%, the flexural modulus significantly improved by about 23.1%. The enhancement in the mechanical properties of the iPP/BA-Al_2_O_3_ nanocomposite is observed, which can be attributed to the improved interactions between iPP and BA-Al_2_O_3_ NPs.

**Fig. 4:**
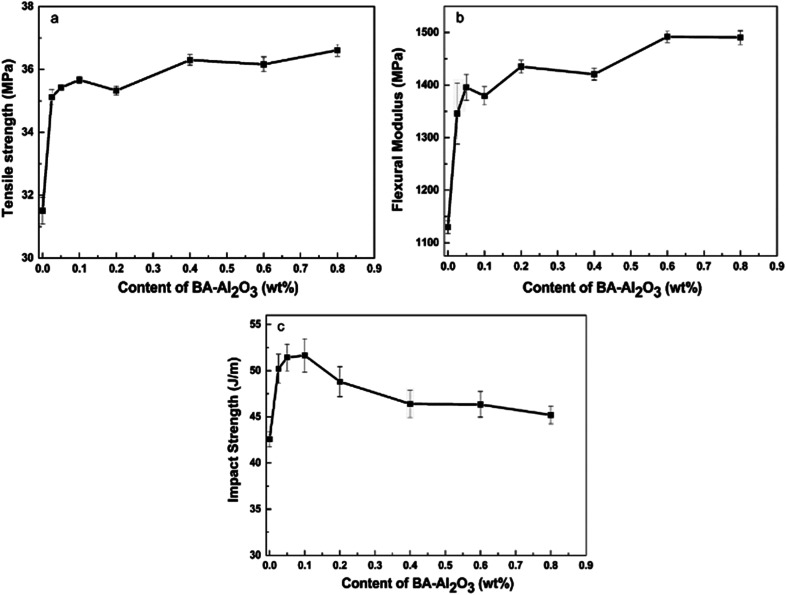
Effects of the mass fraction of BA-Al_2_O_3_ NPs on the (a) tensile strength, (b) flexural modulus, and (c) impact strength of iPP.

Fig. 4 and ESI Table S1† present the mechanical properties of the iPP/BA-Al_2_O_3_ nanocomposites as a function of NPs content. An initial increase in stiffness at low NPs concentrations can be distinguished, which remains constant with the increase in NPs content. An increase of 11.4% in tensile strength and an increase of 19.1% in flexural modulus were achieved in the iPP nucleated by 0.025 wt% BA-Al_2_O_3_. When the content of BA-Al_2_O_3_ is 0.2 wt%, the tensile strength and flexural modulus of the nucleated iPP improved by about 16.2% and 32.0%, respectively, compared with that of pure iPP. Generally, the toughness will show a trend opposing that of the stiffness. However, a great enhancement of more than 20% of the impact strength of the nanocomposites is obtained upon the incorporation of 0.1 wt% BA-Al_2_O_3_. At higher BA-Al_2_O_3_ concentrations, this increase progressively plateaus, but remains higher than that of neat iPP. From these experimental results, it can be concluded that the presence of BA-Al_2_O_3_ has a significant impact on the mechanical performance and maintains a good balance between the stiffness and toughness of iPP nanocomposites.

### 3.3 Effects of interfacial interactions on the mechanical properties of iPP/BA-Al_2_O_3_ nanocomposites

It is known that the interfacial interaction is of great significance in determining the properties of nanocomposites. In general, poor interactions between the NPs and the polymer matrix in nanocomposites will introduce artificial defects, which consequently result in a negative effect on the mechanical properties.^39^ Therefore, investigating the interfacial interactions between iPP and BA-Al_2_O_3_ is considered crucial as the nanocomposites exhibit excellent mechanical properties. The interfacial adhesion between the matrix and the dispersed NPs can be determined by applying the famous Einstein equation, as shown in eqn (1):^40,41^

1 *E*_c_/*E*_m_ = (1 + *BV*_f_)

where *E*_c_ and *E*_m_ are the elastic moduli of the composite and the polymer matrix, respectively. *B* is the parameter characterizing the interfacial adhesion. *V*_f_ is the volume fraction of NPs in the nanocomposites, which can be calculated using eqn (2):

2 *V*_f_ = *ρ*_m_*W*_f_/[(*ρ*_m_ − *ρ*_f_)*W*_f_ + *ρ*_f_]

where *W*_f_ is the weight fraction of NPs, *ρ*_m_ is the density of iPP (0.92 g cm^−3^), and *ρ*_f_ is the density of the Al_2_O_3_ NPs (3.97 g cm^−3^).

**Fig. 5:**
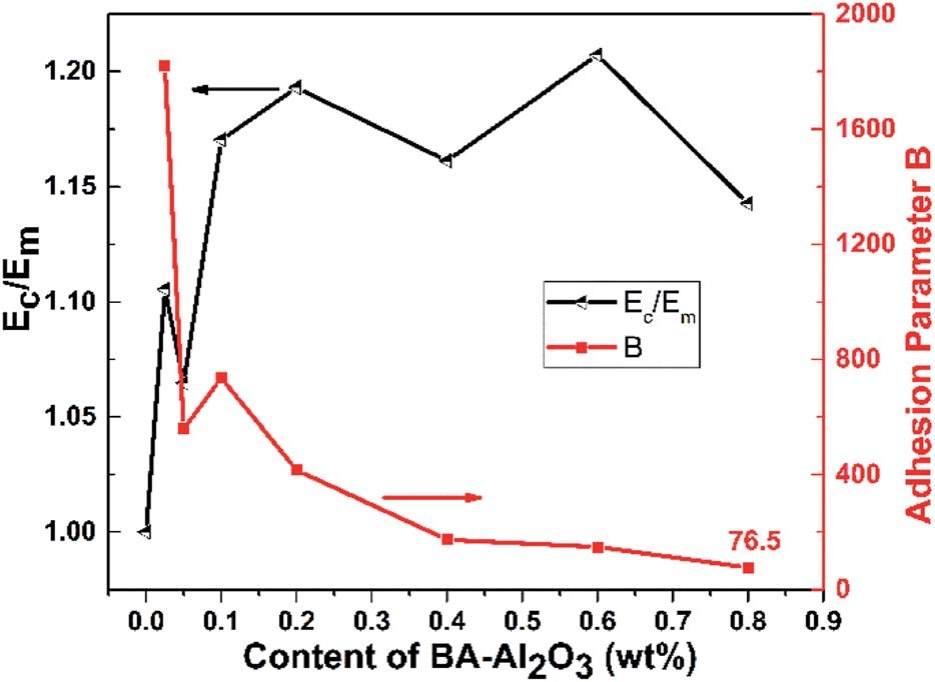
Variation of the relative elastic modulus and adhesion parameter *B* as a function of BA-Al_2_O_3_ NP content.

If there is no adhesion between the matrix and the NPs, *B* becomes 1, while for strong adhesion, *B* takes values higher than 2.5. In Fig. 5, the variation of the relative elastic modulus (see ESI Table S1†) and adhesion parameter *B* (calculated from eqn (1)) with the BA-Al_2_O_3_ NPs content is illustrated. It can be seen clearly that the constant *B* is much higher than 2.5 for the low NPs concentration of 0.025 wt%, indicating that the adhesion between iPP and BA-Al_2_O_3_ is very strong. The low-filled nanocomposites have better dispersed NPs aggregates, which indicates that larger surface is available to contribute to better adhesion.^41^ The parameter decreases as the NPs loading increases, suggesting that aggregation leads to the decrease in the available surface of the BA-Al_2_O_3_ NPs, which prevents good adhesion. However, it is noted that though the adhesion is poorer at high NPs content, the adhesion parameter is still much higher than 2.5, suggesting that the interfacial adhesion is very strong.

The noteworthy interfacial adhesion observed in the nanocomposites with low NPs content can be attributed to the special interfacial interactions. In the present study, the CH–π interaction between the methyl groups group of iPP and the benzene rings of BA-Al_2_O_3_ may play an important role in linking the nanoparticle surface and the polypropylene matrix. This linkage allows the nanocomposites to behave like a unit, in which the robust NPs make the polymer stronger, which is consistent with the fact that good interfacial interaction has a significant effect on the mechanical properties.^17^ Therefore, compared with the as-received Al_2_O_3_ NPs, the simply modified interphase could exhibit higher stiffness and toughness.

### 3.4 Crystallization behaviors of the nanocomposites

**Fig. 6:**
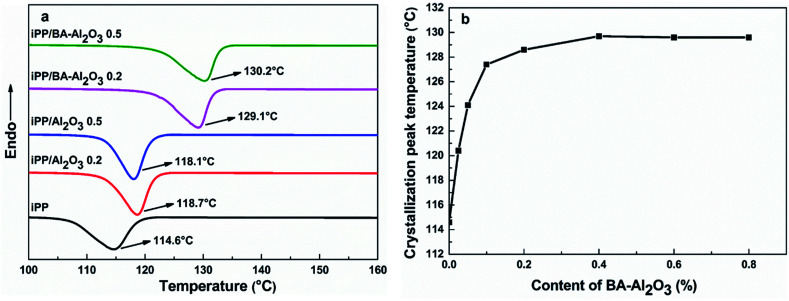
(a) DSC heat flow curves during cooling at a cooling rate of 20 °C min^−1^ for neat iPP and iPP/Al_2_O_3_ and iPP/BA-Al_2_O_3_ nanocomposites at a 0.2 wt% and 0.5 wt% NP loading. (b) Effects of BA-Al_2_O_3_ content on the crystallization behavior of iPP revealed by DSC at a cooling rate of 20 °C min^−1^.

As the nanocomposites exhibit strong interfacial interaction, the effect of the interfacial interaction on the crystallization behavior was also investigated. The crystallization temperature (*T*_C_) is a measure of the crystallization behavior of the polymer upon cooling from the melt. The higher *T*_C_ value suggests higher nucleation efficiency and faster crystallization rate and even shorter progressing times. Fig. 6a shows the DSC heat flow curves of neat iPP and the iPP/Al_2_O_3_ and iPP/BA-Al_2_O_3_ nanocomposites at 0.2 wt% and 0.5 wt% loading during cooling at a cooling rate of 20 °C min^−1^. It is evident from the *T*_C_ values that there is slight improvement upon the incorporation of 0.2 wt% pristine Al_2_O_3_ NPs. With the increase in Al_2_O_3_ NPs content, lower peak temperature is observed. With the addition of only 0.2 wt% BA-Al_2_O_3_ NPs, the *T*_C_ of the iPP/BA-Al_2_O_3_ nanocomposites shows a remarkable increase of ∼15 °C compared to pure iPP, which is comparable to an increase of ∼10 °C for pristine iPP/Al_2_O_3_. Comparison of the nanocomposites at equal NPs loading permits further consideration of the changes in the nucleation ability of the Al_2_O_3_ NPs upon surface modification. These changes can be correlated with changes in the polymer-NPs interfacial interactions.^38^ The enhanced nucleation effects at such low content is not observed in many iPP nanocomposites containing functionalized Al_2_O_3_ NPs,^9,23^ which can be attributed to the special interfacial interaction between iPP and BA-Al_2_O_3_ NPs.

Fig. 6b shows the effect of the BA-Al_2_O_3_ content on the nonisothermal crystallization behavior under a cooling rate of 20 °C min^−1^. It can be seen that compared to pure iPP, the addition of low amounts of BA-Al_2_O_3_ (0.025 wt%) can induce iPP crystallization at higher temperature, suggesting that the addition of BA-Al_2_O_3_ apparently accelerates the crystallization of iPP due to strong heterogeneous nucleation effect in the nanocomposites. With an increase in BA-Al_2_O_3_ NPs content, *T*_C_ significantly increases as expected. Moreover, the iPP with 0.2 wt% BA-Al_2_O_3_ exhibits the greatest enhancement in *T*_C_ of nearly 14 °C. Further increase in the BA-Al_2_O_3_ NPs content can result in a slight improvement in the iPP crystallization ability, and a slight reduction in *T*_C_ eventually occurs when the BA-Al_2_O_3_ NPs content increases to 0.6 wt% and 0.8 wt%. The phenomenon of saturation concentration could be attributed to the decrease in the effective concentration resulting from the agglomeration of additives at high loadings.^42,43^ The addition of BA-Al_2_O_3_ NPs shows two types of effects on the crystallization behavior of iPP. On one hand, the BA-Al_2_O_3_ NPs act as a heterogeneous nucleating agent to increase the crystallization nucleation rate. On the other hand, the addition of higher amounts of BA-Al_2_O_3_ NPs can induce topological confinement effects that can eventually lead to the deterioration of nucleation and crystallization kinetics.^40^ The DSC heat flow curves of the nanocomposites during heating at a rate of 10 °C min^−1^ are presented in ESI Fig. S3 and S4.† All nanocomposites show only one melting peak of 165 °C, which is the characteristic melting temperature of the α-phase of PP. It is clear that the addition of Al_2_O_3_ or BA-Al_2_O_3_ does not change the crystal form of iPP.

**Fig. 7:**
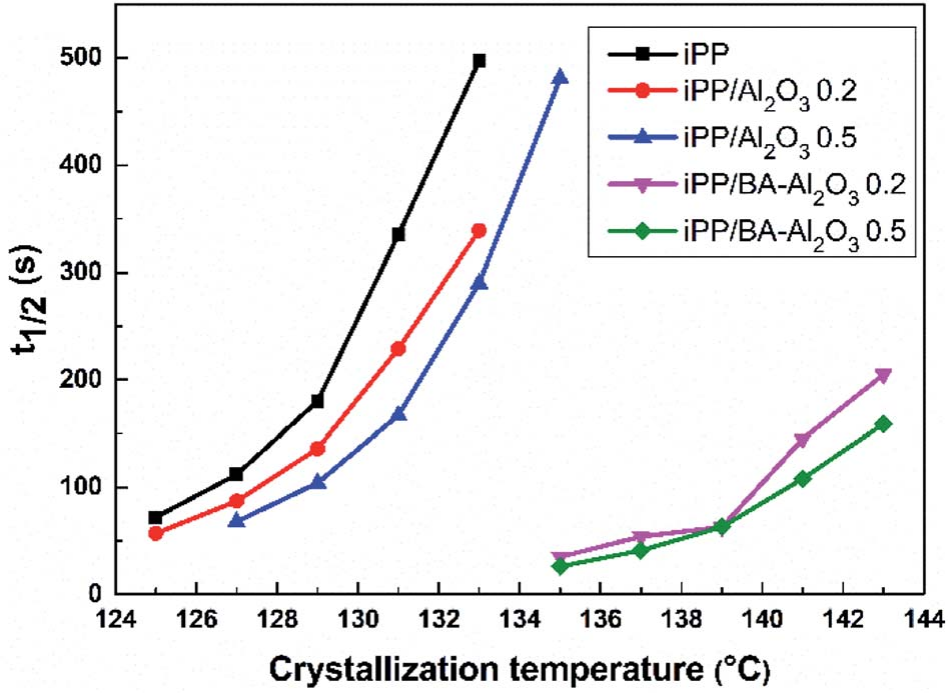
*t*_1/2_ of neat iPP, and iPP/Al_2_O_3_ and iPP/BA-Al_2_O_3_ nanocomposites at 0.2 wt% and 0.5 wt% NPs loading under isothermal crystallization at different temperatures.

Fig. 7 further shows the changes in the crystallization halftime *t*_1/2_ of neat iPP and the iPP/Al_2_O_3_ and iPP/BA-Al_2_O_3_ nanocomposites at 0.2 wt% and 0.5 wt% NPs content under isothermal crystallization at different temperatures. The *t*_1/2_ can be extracted from the DSC measurements to characterize the crystallization rate. It is evident that *t*_1/2_ greatly decreases with the addition of NPs for all nanocomposites, reflecting an enhancement in the crystallization rate of iPP. In addition, shorter *t*_1/2_ at low crystallization temperatures suggests that the nucleating effect is more significant with decreasing crystallization temperature. However, remarkable differences can be observed in the crystallization rate of nanocomposites containing pristine and functionalized Al_2_O_3_ NPs. When the as-received Al_2_O_3_ was added into iPP, the *t*_1/2_ decreased moderately with the increase in Al_2_O_3_ content. The *t*_1/2_ at 131 °C decreases from 5.6 min for pure iPP to 3.8 min and 2.8 min with 0.2 wt% and 0.5 wt% Al_2_O_3_, respectively. On the other hand, the acceleration of the crystallization rate upon addition of 0.2 wt% BA-Al_2_O_3_ is prominent. The *t*_1/2_ decreases to 0.9 min with 0.2 wt% BA-Al_2_O_3_ at 139 °C, whereas the neat iPP and iPP/Al_2_O_3_ nanocomposites have much longer *t*_1/2_ values. As can be clearly seen, the functionalized alumina NPs can promote the crystallization of iPP at much higher temperatures, such as 143 °C, and the crystallization can be completed within 3 min.

**Fig. 8:**
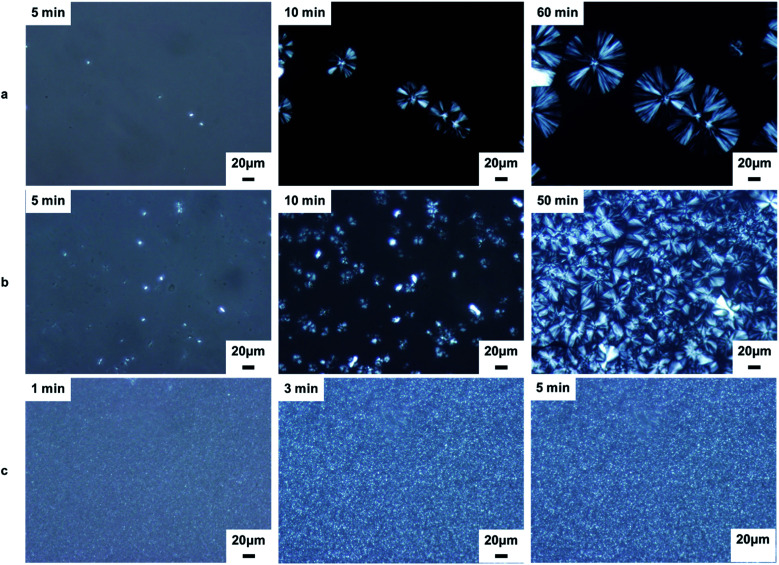
POM micrographs of neat iPP and nanocomposites crystallized during isothermal crystallization at 139 °C (a) iPP; (b) iPP/Al_2_O_3_ 0.2; (c) iPP/BA-Al_2_O_3_ 0.2.

The morphological evolution of the neat iPP and nanocomposites with 0.2 wt% content during isothermal crystallization at 139 °C is revealed by POM photomicrographs, as shown in Fig. 8. The neat iPP exhibits several large spherulites with an average diameter of ∼80 μm after 60 min (Fig. 8a). Upon addition of Al_2_O_3_, the sample (Fig. 8b) exhibited higher nucleation densities (more nuclei) than neat iPP (Fig. 8a) as the crystallization proceeds. The crystallization of iPP/Al_2_O_3_ was almost complete after 50 min with smaller spherulites size (Fig. 8b). With the addition of BA-Al_2_O_3_, the nucleation density increased and the diameter of the spherulites decreased significantly (Fig. 8c). Moreover, the crystallization of the iPP/BA-Al_2_O_3_ nanocomposites was completed in only 3 min (Fig. 8c). It is clear that highly dispersed NPs act as nucleation sites and subsequently accelerate the iPP spherulite growth. With an increase in nuclei density, the spherulites tended to impinge on their neighbors to stop further growth, resulting in smaller spherulites.

Overall, the improvement of the nucleation density observed by POM can be correlated to the acceleration of crystallization, as evidenced by DSC (Fig. 6 and 7). In addition, the sufficient consistency suggests that the CH–π interactions between the polymer and surface of the BA-Al_2_O_3_ NPs contribute greatly to the acceleration of the crystallization rate of iPP/BA-Al_2_O_3_ nanocomposites. Due to the distinct interaction, which may cause the iPP chains to crystallize, the functionalized NPs exhibit more efficient nucleation ability than the pristine Al_2_O_3_ NPs.

### 3.5 Effects on the intrachain conformational ordering

After observing the difference in the nucleation ability of Al_2_O_3_ and BA-Al_2_O_3_, attention was focused on the effect of the interfacial interaction on the physical origin of the strong inducing efficiency of BA-Al_2_O_3_ for iPP crystallization. Till date, different theoretical models have been proposed to describe the progress of polymer crystallization.^44–46^ It is commonly agreed that conformational ordering plays a critical role during this complex process. The molecular chains need to undergo conformational ordering while the persistence length of the helical sequences of the polymer increases before crystallization.^45^ When the length exceeds the critical length, the 3_1_ helical conformation begins to congregate, following which crystallization is induced. Therefore, tracing the intrachain conformational ordering is the best approach to obtain deep insight in the interfacial interaction effect on BA-Al_2_O_3_-driven iPP crystallization.^35^ FTIR is highly sensitive to conformation changes and packing density of molecular chains and was thus used to investigate the crystallization process of the iPP/BA-Al_2_O_3_ nanocomposites.

**Fig. 9:**
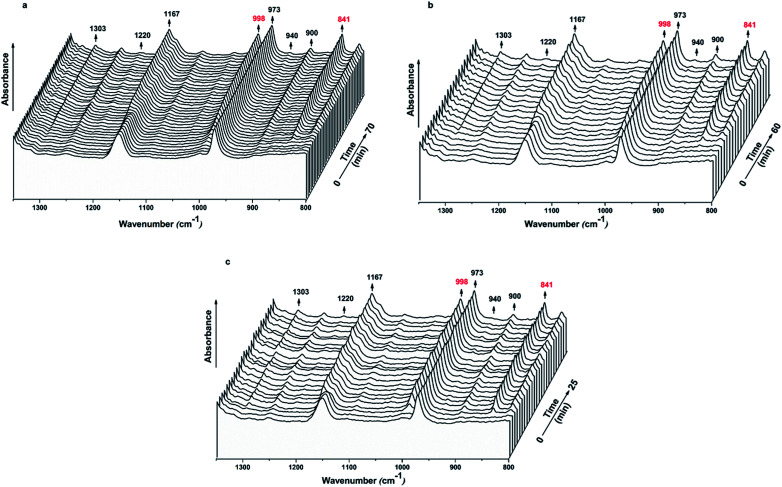
Time-resolved spectra in the 1350–800 cm^−1^ range of (a) iPP, (b) iPP/Al_2_O_3_ 0.2, and (c) iPP/BA-Al_2_O_3_ 0.2 isothermally crystallizing at 139 °C.

Fig. 9 reveals the time-resolved FTIR spectra of iPP, iPP/Al_2_O_3_ 0.2, and iPP/BA-Al_2_O_3_ 0.2 isothermally crystallizing at 139 °C. The intensity of the regularity bands changes as the time increases. The IR bands at 940, 1220, 1303, 1167, 841, 998, 900, and 973 cm^−1^ correspond to the 3_1_ helical structures with decreasing degrees of order.^47^ Because different helical lengths show different IR absorption bands, the gain in conformational ordering bands is directly attributed to the augmentation of the helical population.^48,49^ As shown in Fig. 9, short helical structures already exist in the iPP melt, as evidenced by the strong peak at 973 cm^−1^ (helical length with 3–4 monomers). These short helices undergo propagation and incorporation^50^ to form longer helices. Therefore, the intensity of other IR bands corresponding to long helices also increases with time.

According to the Doi–Edwards's dynamics theory, the critical persistence length of iPP for crystallization transition is 11 monomers in the 3_1_ helical conformation.^47^ The conformational band at 998 cm^−1^ corresponds to 10 monomer units, which is suggested to be more sensitive to the stable-to-unstable transition when crystallization is triggered.^35^ The 998 cm^−1^ band is reasonably chosen to analyse the conformation evolution during crystallization, and the 841 cm^−1^ band corresponding to the helical length with 12 monomers is taken as the crystalline signal.^35,45^ The evolution of the two regular bands may help understand the crystallization process and particularly determine how BA-Al_2_O_3_ accelerates iPP crystallization at the early stages.

**Fig. 10:**
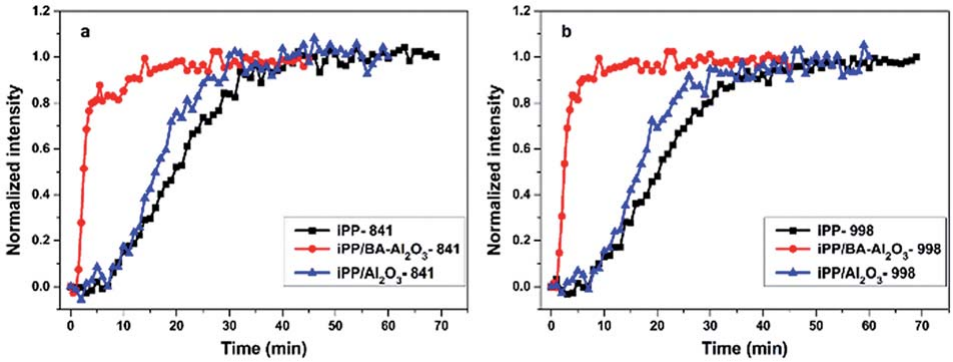
Normalized intensity of the (a) crystalline band at 841 cm^−1^ and (b) conformational ordering band at 998 cm^−1^ as a function of time for iPP, iPP/Al_2_O_3_ 0.2, and iPP/BA-Al_2_O_3_ 0.2 isothermally crystallizing at 139 °C.

Fig. 10a illustrates the normalized intensity of the crystalline band (I841) as a function of crystallization time for iPP and its nanocomposites. The crystallization of iPP is slightly accelerated in the presence of 0.2 wt% Al_2_O_3_, which is consistent with the DSC results. With the introduction of 0.2 wt% BA-Al_2_O_3_, the *t*_1/2_ of the iPP/BA-Al_2_O_3_ 0.2 nanocomposites decreases from 20.3 to 2.5 min. Interestingly, the corresponding variations in the conformational ordering bands follow the same trend as that presented in Fig. 10b. This implies that BA-Al_2_O_3_ also accelerates the conformational ordering of iPP, which is associated with an acceleration of crystallization. The *in situ* FTIR results provided deeper insight in BA-Al_2_O_3_-driven polymer crystallization by establishing a relationship with the BA-Al_2_O_3_ induced conformation ordering. Upon addition of BA-Al_2_O_3_, the short helical segments tend to propagate or incorporate to form long helical segments due to the interfacial interactions between the NPs surface and the polymer.

## 4. Discussions

The experimental results clearly demonstrate that the benzoic acid functionalized alumina NPs (BA-Al_2_O_3_ NPs) can enhance both the mechanical performance and the crystallization behavior. The reaction of benzoic acid with alumina NPs did not change the crystal structure (see ESI Fig. S2†). The NPs surfaces were covalently bonded to benzene rings after functionalization. Thus, it is supposed that special interactions are formed between the polymer and the benzene rings of BA-Al_2_O_3_.

The CH–π interaction between carbon-hydrogen groups (CH groups) and π-systems has been known for many years. Although the strength of the CH–π interaction is only one-tenth of a hydrogen bond, the interaction still remarkably influences polymer nanocomposites.^29,30,51–53^ Considering the aromatic rings as π-systems and iPP as ‘CH-rich’ polymers, CH–π interactions should occur between BA-Al_2_O_3_ and iPP, which improves the conformational ordering and crystallization kinetics as well as the mechanical properties.

**Fig. 11:**
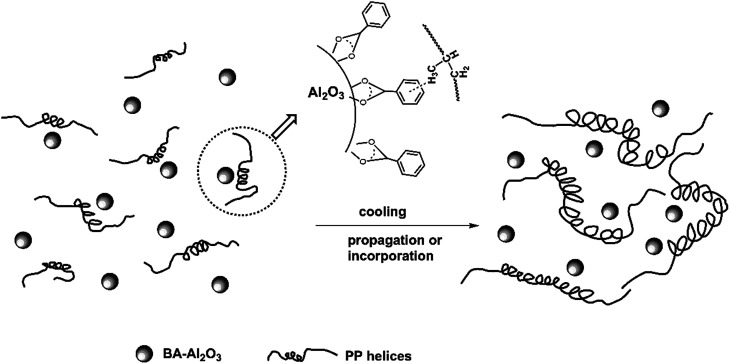
Schematic diagram of intrachain conformation ordering in iPP induced by the CH–π interaction between the polymer and BA-Al_2_O_3_.

The possible mechanism of BA-Al_2_O_3_-induced intrachain conformational ordering is schematically illustrated in Fig. 11. At the initial stage of crystallization, the short helices tend to adsorb on the surface of BA-Al_2_O_3_*via* CH–π interactions, as presented in Fig. 11. It should be mentioned that the interactions occur between the protruding methyl groups and the aromatic rings.

Another study also suggested that intrachain conformational ordering is enhanced by the interaction between the protruding methyl groups of iPP and graphene layers of sp^2^-bonded carbon.^54^ These specific type of CH–π interaction has not yet been proved experimentally. Short helices undergo propagation and incorporation to form long helical segments during the cooling process.^50^ Then, the preexisting long helices pack into the formed coupled helices, thus significantly accelerating crystallization (see Fig. 11).

In summary, CH–π interaction plays a critical role in reducing the free energy barriers of nucleation and subsequent crystallization and growth. The mechanical properties are believed to be affected by this interaction as well. Some studies have reported that the CH stretching frequency of polymers in the FTIR spectrum will shift to lower frequencies due to of CH–π interactions.^55^ However, the FTIR spectrum recorded in this study does not exhibit shifts of the CH stretching frequency of the methine (*ν*CH), methylene (*ν*CH_2_), and methyl (*ν*CH_3_) groups of iPP. Unfortunately, the existence of CH–π interaction cannot be proved directly. However, the present experimental results clearly provide circumstantial evidence, implying the presence of CH–π interactions.

## 5. Conclusions

PP nanocomposites with small amounts of benzoic acid-functionalized Al_2_O_3_ NPs were prepared by melt-mixing and were found to have greatly enhanced mechanical properties and crystallization behaviors. The tensile strength, flexural modulus, and impact strength were significantly increased in the iPP/BA-Al_2_O_3_ nanocomposites compared to those of the iPP/Al_2_O_3_ nanocomposites and pristine iPP. According to the Einstein equation, the interfacial adhesion was estimated by the parameter *B*. The values of constant *B* are much higher than 2.5, indicating that the interfacial adhesion is significantly strong. With the addition of 0.2 wt% BA-Al_2_O_3_ NPs, the crystallization temperature remarkably increases by 14.8 °C. Pristine Al_2_O_3_ only showed slight nucleation ability at the same content. Moreover, the crystallization rate of iPP significantly increased upon incorporating the BA-Al_2_O_3_ NPs. Finally, the dynamic process of BA-Al_2_O_3_-induced iPP crystallization was investigated at the molecular level using time-resolved FTIR measurements. The presence of BA-Al_2_O_3_ facilitates the conformational ordering as well as the formation of long helical segments in the initial crystallization stage. It was supposed that the CH–π interaction between the protruding methyl groups of iPP and the benzene rings of BA-Al_2_O_3_ accelerates the intrachain conformational ordering, which is accompanied by a considerable enhancement of the crystallization. Subsequently, the mechanical properties of the products also improved remarkably due to the interfacial interactions.

## Conflicts of interest

There are no conflicts to declare.

## Supplementary Material

RA-008-C8RA01069B-s001
