# Supplementary material for: Effect of benzoic acid surface modified alumina nanoparticles on the mechanical properties and crystallization behavior of isotactic polypropylene nanocomposites
Source: RSC Adv. 2018 Jun 6;8(37):20790–800. doi: 10.1039/c8ra01069b (PMC9080881; doi:10.1039/c8ra01069b)
Supplement: RA-008-C8RA01069B-s001 [file RA-008-C8RA01069B-s001.pdf]

Electronic Supplementary Material (ESI) for RSC Advances.
This journal is © The Royal Society of Chemistry 2018

Supporting Information

The Effect of Benzoic Acid Surface Modified Alumina Nanoparticles on the Crystallization Behavior and Mechanical Properties of Isotactic Polypropylene Nanocomposites

Xiaofeng Jiang,[a] Wenxue Zhang,[b] Shicheng Zhao,[a] Shuai Zhou,[a] YaoQi Shi[a] and Zhong Xin*[a]

[a]Shanghai Key Laboratory of Multiphase Materials Chemical Engineering, State-Key Laboratory of Chemical Engineering, East China University of Science and Technology, Shanghai, 200237, China. E-mail: xzh@ecust.edu.cn

[b]Lanzhou Petrochemical Research Center, PetroChina, 730060, China

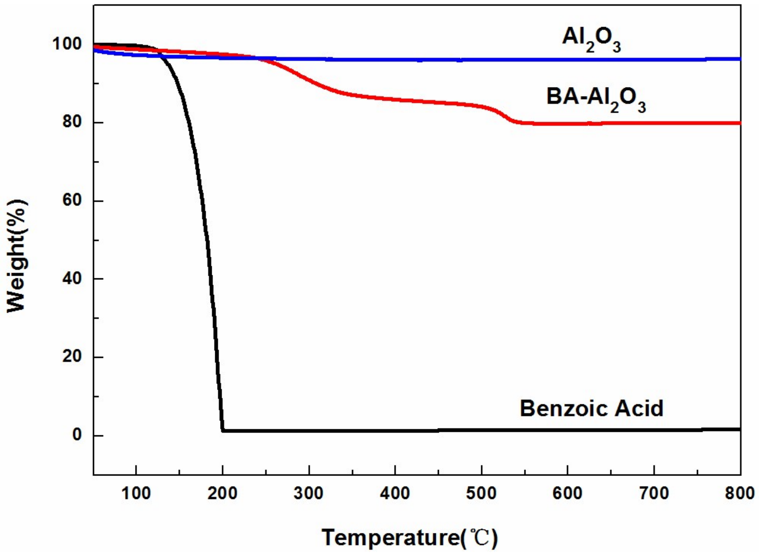

Fig. S1 TGA of pristine alumina NPs, functionalized alumina NPs and native carboxylic acids

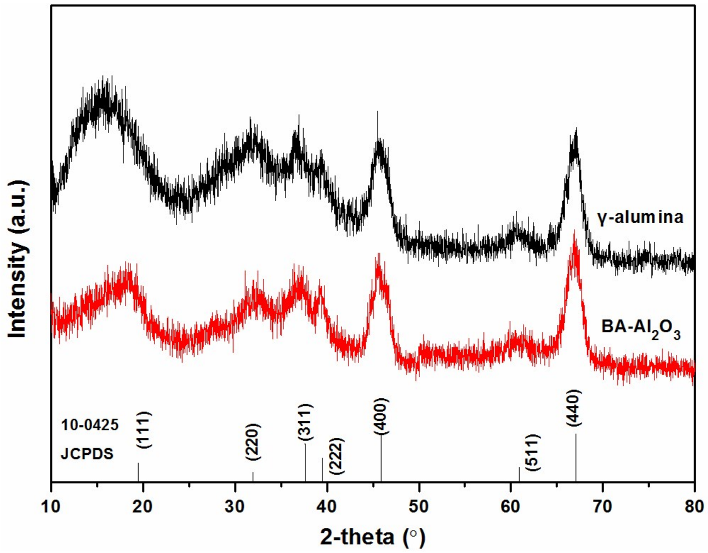

Fig. S2 WAXD patterns of pristine alumina NPs and functionalized alumina NPs

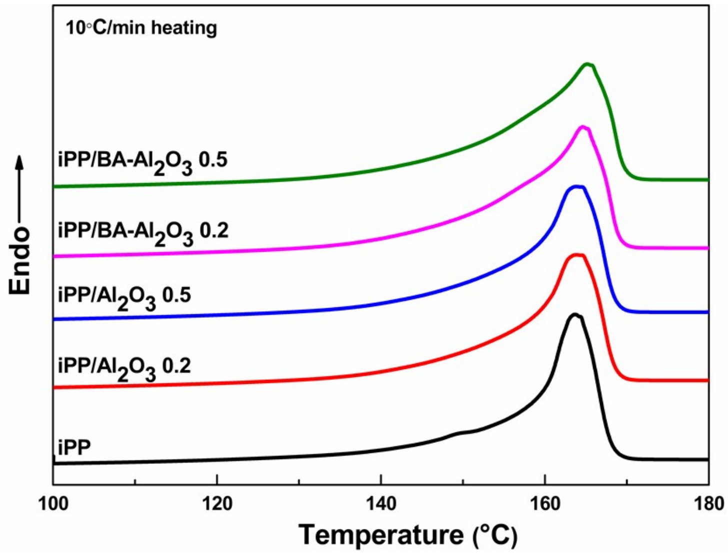

Fig. S3 The DSC heat flow curves during heating at a rate of 10 °C/min for neat iPP, iPP/Al_2O_3 and iPP/BA-Al_2O_3 nanocomposites at a 0.2wt% and 0.5wt% NPs loading

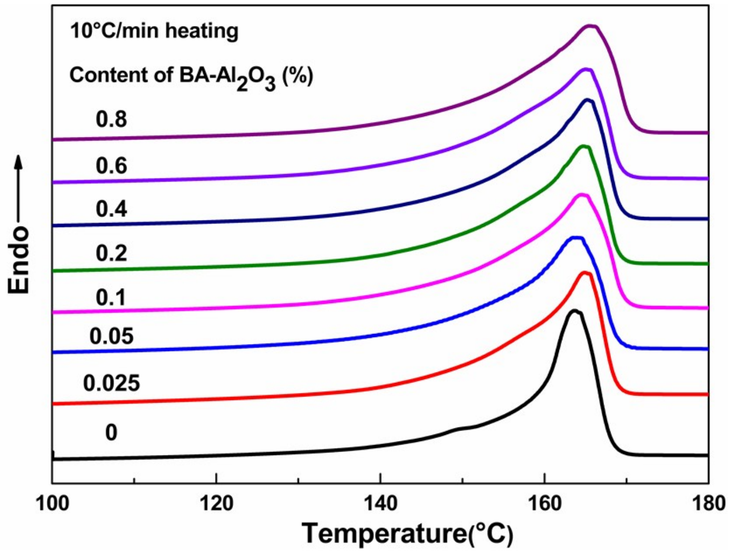

Fig. S4 The DSC heat flow curves during heating at a rate of 10 °C/min for iPP/BA-Al_2O_3 nanocomposites at different loading

Table S1 Mechanical Properties of the Prepared Nanocomposites

$BA\text{-}Al_2O_3$, wt %	elastic modulus, MPa	stress at yield, MPa	tensile strength, MPa	flexural modulus, MPa	impact strength, J/m
0	290 ± 9	1369 ± 18	31.5 ± 0.4	1129 ± 12	42.6 ± 0.8
0.025	320 ± 19	1526 ± 11	35.1 ± 0.2	1345 ± 58	50.2 ± 2.6
0.05	309 ± 12	1539 ± 3	35.4 ± 0.1	1395 ± 25	51.4 ± 1.4
0.1	339 ± 13	1550 ± 5	35.7 ± 0.1	1379 ± 17	51.6 ± 1.8
0.2	346 ± 61	1535 ± 6	35.3 ± 0.1	1434 ± 12	48.8 ± 1.6
0.4	337 ± 16	1545 ± 7	36.3 ± 0.2	1420 ± 11	46.4 ± 3.5
0.6	350 ± 61	1539 ± 10	36.2 ± 0.2	1491 ± 11	50.9 ± 2.4
0.8	331 ± 30	1558 ± 8	36.6 ± 0.2	1490 ± 13	45.2 ± 0.9
